# Supplementary material for: Tracing the oxygen isotope composition of the upper Earth's atmosphere using cosmic spherules
Source: Nat Commun. 2017 Jun 1;8:15702. doi: 10.1038/ncomms15702 (PMC5461487; doi:10.1038/ncomms15702)
Supplement: Supplementary Information — Supplementary Tables [file ncomms15702-s1.pdf]

Supplementary Table 1 | Oxygen isotope data of air molecular oxygen from two different campaigns. The samples from D1 to ST05 were extracted using an early protocol. The samples from B1 to BP2 were extracted with an improved protocol. The values are reported on VSMOW2-SLAP2 scale.

**Sample**	**δ^{17}O (‰)**	**δ^{18}O (‰)**	**Δ'^{17}O (‰)**
D1	12.12 ± 0.08	23.87 ± 0.16	-0.469 ± 0.012
D2	12.21 ± 0.08	24.01 ± 0.16	-0.448 ± 0.012
D3	12.11 ± 0.08	23.83 ± 0.16	-0.458 ± 0.012
D4	12.28 ± 0.08	24.15 ± 0.16	-0.459 ± 0.012
DAr1	12.03 ± 0.08	23.67 ± 0.16	-0.457 ± 0.012
DAr2	12.03 ± 0.08	23.70 ± 0.16	-0.470 ± 0.012
DAr3	12.27 ± 0.08	24.19 ± 0.16	-0.479 ± 0.012
DAr4	12.05 ± 0.08	23.76 ± 0.16	-0.483 ± 0.012
ST02	12.16 ± 0.08	23.98 ± 0.16	-0.479 ± 0.012
ST03	12.18 ± 0.08	24.00 ± 0.16	-0.477 ± 0.012
ST04	12.16 ± 0.08	23.96 ± 0.16	-0.473 ± 0.012
ST05	12.14 ± 0.08	23.90 ± 0.16	-0.466 ± 0.012
Mean	12.15 ± 0.02	23.93 ± 0.04	-0.470 ± 0.003
B01	12.22 ± 0.06	24.08 ± 0.13	-0.477 ± 0.019
B02	12.21 ± 0.06	24.07 ± 0.13	-0.482 ± 0.019
B07	12.26 ± 0.06	24.15 ± 0.13	-0.474 ± 0.019
B08	12.33 ± 0.06	24.22 ± 0.13	-0.443 ± 0.019
B09	12.38 ± 0.06	24.43 ± 0.13	-0.501 ± 0.019
B11	12.22 ± 0.06	24.02 ± 0.13	-0.445 ± 0.019
BP1	12.25 ± 0.06	24.11 ± 0.13	-0.462 ± 0.019
BP2	12.26 ± 0.06	24.14 ± 0.13	-0.469 ± 0.019
Mean	12.27 ± 0.02	24.15 ± 0.05	-0.469 ± 0.007

Supplementary Table 2 | Mass and oxygen and iron isotopic compositions of analyzed I-type spherules aliquots and oxygen isotope composition of high-T oxidation experiments H2 - H4. The oxygen isotope data are reported relative to VSMOW2 scale. The iron isotope data are reported relative to IRMM-014 (*repeated measurements on an individual aliquot).

**Sample**	**Mass (µg)**	**δ^{17}O (‰)**	**δ^{18}O (‰)**	**Δ'^{17}O (‰)**	**δ^{56}Fe (‰)**	**δ^{57}Fe (‰)**
32-1	52	18.6	36.4	-0.57	30.15*	45.02*
- " -	38	18.6	36.5	-0.61	30.26*	45.23*
- " -	30	18.0	35.4	-0.63		
- " -	29	17.2	33.8	-0.56		
- " -	47	21.6	42.3	-0.64		
- " -	36	20.4	40.1	-0.67		
- " -	27	20.0	39.3	-0.65		
- " -	29	19.8	38.9	-0.63		
Mean		19.3 ± 0.5	37.8 ± 1.0	-0.62 ± 0.01	30.21 ± 0.17	45.13 ± 0.31
32-2	29	21.8	42.9	-0.73	30.91*	47.76*
- " -	39	22.6	44.5	-0.75	30.92*	47.74*
- " -	27	20.1	39.7	-0.75		
- " -	27	22.2	43.6	-0.70		
- " -	20	21.4	42.1	-0.68		
Mean		21.6 ± 0.5	42.5 ± 0.8	-0.72 ± 0.02	30.92 ± 0.02	47.75 ± 0.03
37-2	30	18.9	37.4	-0.75	22.34*	33.38*
- " -	27	18.7	37.1	-0.77	22.29*	33.34*
- " -	21	17.8	35.3	-0.70	22.19*	33.32*
- " -	26	17.2	33.9	-0.66		
Mean		18.2 ± 0.4	35.9 ± 0.8	-0.72 ± 0.02	22.27 ± 0.15	33.35 ± 0.05
7m-01	28	19.3	38.0	-0.63	23.29*	34.81*
- " -	42	19.9	39.4	-0.74	23.28*	34.78*
- " -	31	20.7	40.9	-0.82		

- " -	32	21.9	43.4	-0.84		
Mean		20.5 ± 0.5	40.4 ± 1.1	-0.76 ± 0.04	23.29 ± 0.02	34.79 ± 0.05
**Sample**	***T* (°C)**	**δ^{17}O (‰)**	**δ^{18}O (‰)**	**Δ'^{17}O (‰)**		
H2	1510 °C	10.36	20.38	-0.40		
H3	1550 °C	10.21	19.97	-0.33		
H4	1590 °C	10.35	20.31	-0.37		
Mean		10.31 ± 0.03	20.22 ± 0.04	-0.36 ± 0.0		

Supplementary Table 3 | Calculated degree of evaporation f, evaporative increase in $\delta^{18}O$ and reconstructed $\delta^{18}O$ of the I-type spherules after oxidation, but before evaporative enrichment in ^{18}O and reconstructed $\Delta'^{17}O$ of upper mesospheric oxygen with the respective 1σ uncertainties.

Parameter	32-01	32-02	37-02	7m-01
$f_{evaporated}$ (from $\delta^{56}Fe$ and Eqn. 1)	0.81	0.82	0.70	0.73
Initial diameter (oxide, µm)	950	800	630	640
Increase in $\delta^{18}O$ during evaporation (‰)	36 ‰	37 ‰	16 ‰	28 ‰
$\delta^{18}O_{Fe,Ni\ oxide}$ (‰, prior to evaporation)	2 ‰	5 ‰	10 ‰	12 ‰
$\delta^{18}O_{Fe,Ni\ oxide} - \delta^{18}O_{air}$	-22 ‰	-19 ‰	-14 ‰	-12 ‰
$\Delta'^{17}O_{air}$ (proxy) (‰)	-0.43 ± 0.07	-0.420 ± 0.07	-0.51 ± 0.06	-0.47 ± 0.07

Supplementary Table 4 | Mass, size, volume, and calculated density of the studied spherules.

Sample	Mass (mg)	Diameter (μm)	Volume (10^{-6} cm^3)	Density (g cm^{-3})
32-01	366 ± 5	548 ± 25	86 ± 12	4.3 ± 0.6
32-02	212 ± 5	453 ± 10	49 ± 3	4.4 ± 0.4
37-01	185 ± 5	419 ± 5	39 ± 1	4.8 ± 0.3
7m-01	159 ± 5	409 ± 5	36 ± 1	4.4 ± 0.3

Supplementary Table 5 | Monte Carlo model parameters.

Parameter	Value	1σ
$\delta^{18}O$ of spherule	measured	0.9 ‰
$\Delta'^{17}O$ of spherule	measured	0.01 ‰
$\delta^{56}Fe$ of spherule	measured	0.17 ‰
Evaporation $\delta^{18}O$ vs. $\delta^{56}O$ slope (Fig. 2)	1.18	0.05
$\theta_{evaporation}$	0.5096	0.0005
$\theta_{oxidation}$	0.506	0.003
$\delta^{18}O_{oxygen}$ (target value)	24	0.2
